# Ampliando Perspectivas sobre a Adequação do Cardioversor Desfibriladors Implantável: Papel dos Marcadores Econômicos e Psicossociais, Subtipos de Miocardiopatia e Terapias Avançadas

**DOI:** 10.36660/abc.20250066

**Published:** 2025-05-29

**Authors:** Ömer Faruk Yılmaz, Yusuf Ziya Şener

**Affiliations:** 1 Afyonkarahisar Health Sciences University Department of Cardiology Afyonkarahisar Turquia Afyonkarahisar Health Sciences University - Department of Cardiology, Afyonkarahisar – Turquia; 2 Erasmus MC Thoraxcentrum Cardiology Department Rotterdam ZH Holanda Erasmus MC Thoraxcentrum - Cardiology Department, Rotterdam, ZH – Holanda

**Keywords:** Cardiomiopatias, Insuficiência Cardíaca, Terapêutica, Desfibriladores, Mortalidade

**Caro Editor,**

Lemos com grande interesse o artigo de Carvalho et al.intitulado "Implantes Potencialmente Inapropriados de Cardioversor-Desfibrilador na Prevenção Secundária de Morte",^1^ que destaca o papel crítico dos marcadores socioeconômicos e psicossociais (MESP) na predição da mortalidade precoce pós-implante de cardioversor desfibrilador implantável (CDI). Embora o estudo forneça insights valiosos sobre avaliações de equipe multidisciplinar (EMD), gostaríamos de abordar duas limitações significativas que podem refinar pesquisas futuras e a prática clínica.

O estudo enfatiza corretamente as implicações prognósticas do MESP, mas não aborda o papel das terapias médicas orientadas por diretrizes (TOPD), que demonstraram reduzir significativamente o risco de morte cardíaca súbita em pacientes com insuficiência cardíaca com fração de ejeção reduzida (ICFEr). Por exemplo, os inibidores do cotransportador de sódio-glicose-2 (SGLT2), como a dapagliflozina, reduzem a mortalidade cardiovascular em 14% (razão de risco [RR]: 0,86; intervalo de confiança [IC] de 95%: 0,76–0,98), conforme demonstrado no estudo DAPA-HF,^2^ independente da fração de ejeção do ventrículo esquerdo (FEVE). Da mesma forma, o antagonista do receptor mineralocorticoide (ARM) espironolactona reduziu significativamente a mortalidade por todas as causas em 30% (razão de risco [RR]: 0,70; IC de 95%: 0,60–0,82; p<0,001) em pacientes com ICFEr avançada no estudo RALES,^3^ independentemente de fatores socioeconômicos. Além disso, a terapia de ressincronização cardíaca demonstrou melhorar a sobrevida em pacientes com duração do QRS >150 mse bloqueio do ramo esquerdo, reduzindo a mortalidade em 36% em comparação ao CDI sozinho.^4^ A falta de dados sobre a adesão à TOPD e elegibilidade à CRT no estudo limita a capacidade de discernir completamente a significância prognóstica independente do MESP. Estratificar os resultados pela utilização da TOPD e pela responsividade à CRT poderia fornecer uma compreensão mais matizada da interação entre variáveis socioeconômicas, psicossociais e clínicas.

Outra limitação crítica é a heterogeneidade dos subtipos de cardiomiopatia. A coorte incluiu 51% dos pacientes com cardiomiopatia de Chagas, mas não analisou etiologias de alto risco (por exemplo, amiloidose cardíaca, sarcoidose, cardiomiopatias genéticas malignas) separadamente. A amiloidose transtirretina (ATTR-CM), por exemplo, tem uma sobrevida mediana de 3,6 anos após o diagnóstico, com uma alta carga arrítmica, apesar dos CDIs.^5^ Por outro lado, a cardiomiopatia periparto e a miocardite frequentemente apresentam recuperação do VE (40–50% dos casos), tornando o implante precoce do CDI potencialmente inapropriado.^6 , 7^ A cardiomiopatia chagásica envolve uma fisiopatologia única, dominada por fibrose e disfunção autonômica, o que pode confundir os resultados relacionados ao MESP.^8^ Análises de subgrupos por etiologia podem esclarecer se o MESP afeta todas as populações igualmente ou interage com fatores específicos da doença.

Finalmente, a discrepância entre a taxa de mortalidade prevista em um ano (15,9%) e a taxa observada (25,8%) com base no escore de risco MAGGIC, destaca a influência potencial de variáveis não contabilizadas, como adesão à TOPD, carga arrítmica ou outros fatores de risco. Além disso, a alta prevalência da doença de Chagas na coorte do estudo levanta preocupações sobre a generalização desses achados para regiões não endêmicas. Embora o estudo de Carvalho et al.^1^ enfatize o valor das avaliações de EMD, a integração de dados sobre adesão à TOPD, elegibilidade para CRT e estratificação por subtipo de cardiomiopatia em pesquisas futuras pode melhorar a precisão dos modelos prognósticos e aprimorar as estratégias de seleção de pacientes, particularmente em ambientes com recursos limitados. Estudos multicêntricos projetados para abordar essas limitações são cruciais para otimizar os resultados dos pacientes e refinar as práticas de implantação de CDI.
