# Supplementary material for: Ampliando Perspectivas sobre a Adequação do Cardioversor Desfibriladors Implantável: Papel dos Marcadores Econômicos e Psicossociais, Subtipos de Miocardiopatia e Terapias Avançadas
Source: Arq Bras Cardiol. 2025 May 29;122(5):e20250066. [Article in Portuguese] doi: 10.36660/abc.20250066 (PMC12212370; doi:10.36660/abc.20250066)
Supplement: Translation

# Translation: Broadening Insights into Implantable Cardioverter Defibrillator Appropriateness: The Role of Economic and Psychosocial Markers, Cardiomyopathy Subtypes, and Advanced Therapies

**DOI:** 10.36660/abc.20250066i

**Authors:** Ömer Faruk Yılmaz, Yusuf Ziya Şener

**Affiliations:** 1 Afyonkarahisar Health Sciences University Department of Cardiology Afyonkarahisar Türkiye Afyonkarahisar Health Sciences University - Department of Cardiology, Afyonkarahisar – Türkiye; 2 Erasmus MC Thoraxcentrum Cardiology Department Rotterdam ZH Netherlands Erasmus MC Thoraxcentrum - Cardiology Department, Rotterdam, ZH – Netherlands

**Keywords:** Cardiomyopathies, Heart Failure, Therapeutics, Defibrillators, Mortality

**Dear Editor,**

We read with great interest the article by Carvalho et al. titled "Potentially Inappropriate Cardioverter Defibrillator Implants in Secondary Prevention of Death",^1^which highlights the critical role of socioeconomic and psychosocial markers (EPSM) in predicting early mortality post-implantable cardioverter defibrillator (ICD) implantation. While the study provides valuable insights into multidisciplinary team (MDT) assessments, we would like to address two significant limitations that could refine future research and clinical practice.

The study rightly emphasizes the prognostic implications of EPSM but does not address the role of guideline-directed medical therapies (GDMT), which have been shown to significantly reduce the risk of sudden cardiac death in patients with heart failure with reduced ejection fraction (HFrEF). For instance, sodium-glucose co-transporter-2 (SGLT2) inhibitors, such as dapagliflozin, reduce cardiovascular mortality by 14% (hazard ratio [HR]: 0.86; 95% confidence interval [CI]: 0.76–0.98), as demonstrated in the DAPA-HF trial,^2^independent of left ventricular ejection fraction. Similarly, the mineralocorticoid receptor antagonist spironolactone significantly decreased all-cause mortality by 30% (risk ratio [RR]: 0.70; 95% CI: 0.60–0.82; p<0.001) in patients with advanced HFrEF in the RALES trial,^3^irrespective of socioeconomic factors. Additionally, cardiac resynchronization therapy (CRT) has been shown to improve survival in patients with a QRS duration >150 ms and left bundle branch block (LBBB), reducing mortality by 36% compared to ICD alone.^4^The lack of data on GDMT adherence and CRT eligibility in the study limits the ability to discern EPSM's independent prognostic significance fully. Stratifying outcomes by GDMT utilization and CRT responsiveness could provide a more nuanced understanding of the interplay between socioeconomic, psychosocial, and clinical variables.

Another critical limitation is the heterogeneity of cardiomyopathy subtypes. The cohort included 51% of Chagas cardiomyopathy patients but did not analyze high-risk etiologies (e.g., cardiac amyloidosis, sarcoidosis, malignant genetic cardiomyopathies) separately. Transthyretin amyloidosis (ATTR-CM), for instance, has a median survival of 3.6 years post-diagnosis, with a high arrhythmic burden despite ICDs.^5^Conversely, peripartum cardiomyopathy and myocarditis often show LV recovery (40–50% of cases), making early ICD implantation potentially inappropriate.^6 , 7^Chagas cardiomyopathy involves a unique pathophysiology dominated by fibrosis and autonomic dysfunction, which may confound EPSM-related outcomes.^8^Subgroup analyses by etiology could clarify whether EPSM impacts all populations equally or interacts with disease-specific factors.

Finally, the discrepancy between the predicted one-year mortality rate (15.9%) and the observed rate (25.8%) based on the MAGGIC risk score highlights the potential influence of unaccounted variables, such as adherence to GDMT, arrhythmic burden, or other risk factors. Additionally, the high prevalence of Chagas disease within the study cohort raises concerns about the generalizability of these findings to non-endemic regions. While Carvalho et al.^1^study emphasizes the value of MDT assessments, integrating data on GDMT adherence, CRT eligibility, and stratification by cardiomyopathy subtype in future research could improve the accuracy of prognostic models and enhance patient selection strategies, particularly in resource-constrained settings. Multicenter studies designed to address these limitations are crucial for optimizing patient outcomes and refining ICD implantation practices.
