# Supplementary material for: Ampliando Perspectivas sobre a Adequação do Cardioversor Desfibriladors Implantável: Papel dos Marcadores Econômicos e Psicossociais, Subtipos de Miocardiopatia e Terapias Avançadas
Source: Arq Bras Cardiol. 2025 May 29;122(5):e20250066. [Article in Portuguese] doi: 10.36660/abc.20250066 (PMC12212370; doi:10.36660/abc.20250066)
Supplement: Related texts

# Reply: Carta-resposta

**Authors:** William Neves Carvalho

**Affiliations:** 1 Universidade Federal da Bahia Salvador BA Brasil Universidade Federal da Bahia, Salvador, BA – Brasil

De antemão ficamos gratos pelas ponderações e críticas pertinentes e construtivas feitas ao nosso trabalho.^1^

Algumas das limitações relatadas e citadas na carta foram, ainda que de forma sutil, informada pelos autores do artigo.

Outras indagações referentes à comparação (Teste T de Student não pareado) entre os grupos referentes à terapia farmacológica e às etiologias da insuficiência cardíaca estão disponíveis na Tabela 1 .

**Tabela 1:** Comparação entre os grupos COM MEPS vs. SEM MEPS quanto a terapia medicamentosa e etiologias dos pacientes submetidos a implante de CDI como prevenção secundária no Hospital Ana Nery

	Com MEPS	Sem MEPS	
	167	89	p
IECA/BRA/Sacubitril-valsartana	122 (73%)	67 (75%)	0,831
Betabloqueador, n (%)	127 (76%)	76 (85,4%)	0,562
Espironolactona, n (%)	100 (60%)	47 (52,8%)	0,481
ISGLT2, n (%)	14 (8,3%)	11(12,3%)	0,323
Amiodarona, n (%)	99 (59,3%)	46 (51,7%)	0,761
Miocardite, n (%)	18(10,7%)	9 (10,1%)	0,892
Dilatada idiopática	8 (4,8%)	7 (7,9%)	0,126
Amiloidose	7 (4,2%)	5 (5,6%)	0,332
Periparto	2 (1,2%)	4 (4,5%)	0,089
Miocárdio não compactado, n (%)	2 (1,2%)	2 (2,2%)	0,112

IECA: inibidor da enzima conversora da angiotensina; BRA: bloqueador do receptor da aldosterona.

Na Tabela 2 está descrita a quantidade de pacientes por grupo (COM MEPS e SEM MEPS) que evoluíram a óbito no primeiro ano do implante de acordo com cada etiologia.

**Tabela 2:** Óbitos < 1 ano dos pacientes COM MEPS vs.SEM MEPS de acordo com a etiologia da IC que foram submetidos a implante de CDI como prevenção secundária de morte subida cardíaca no Hospital Ana Nery

	Óbitos com MEPS	Sem MEPS
	10/167 (6%)	2/89 (2,2%)
Miocardite	4 (40%)	─
Amiloidose	1 (10%)	1 (50%)
Dilatada idiopática	3 (30%)	1 (50%)
Miocárdio não compactado	2 (20%)	─

Acreditamos que alguns fatores estejam associados à baixa taxa de pacientes em uso de ISGLT2 na amostra, podemos citar alguns fatos: nosso estudo foi iniciado 2,5 anos antes (2017) da publicação do estudo *Dapagliflozin in Patients with Heart Failure and Reduced Ejection Fraction* e da liberação para a comercialização da droga, o qual foi publicado em 2019. Outro fator importante abordado no artigo é a baixa renda mensal familiar da amostra, e o custo do medicamento elevado para o perfil da população estudada, tal fato pode ter contribuído para uma das causas de baixa adesão medicamentosa (um dos marcadores econômicos e psicossociais – MEPS). Vale ressaltar ainda que, até a finalização do seguimento do estudo não haviam programas para facilitação ao acesso gratuito ao ISGLT2 em tempo necessário que pudesse eventualmente aumentar a taxa de uso dos ISGLT2 em tempo hábil para análise dos dados.

Salientamos que apesar da relevância dos fármacos na redução de mortalidade de pacientes com IC, tais variáveis não foram disponibilizadas na versão original do artigo, no entanto acreditamos não ter sido um fator preponderante para mudança do desfecho estudado.

Por fim, não há interpelações e questionamentos sobre os potenciais benefícios da terapia de ressincronização cardíaca (TRC), haja vista que o nosso estudo não abordou essa prótese.

Mantenho-me a disposição para elucidar demais dúvidas

Willliam N. Carvalho

## References

[1] Carvalho WN, Viana TT, Figueiredo CS, Martins F, Passos LCS (2024). Potentially Inappropriate Cardioverter Defibrillator Implants in Secondary Prevention of Death. Arq Bras Cardiol.

# Reply

**Authors:** William Neves Carvalho

**Affiliations:** 1 Universidade Federal da Bahia Salvador BA Brazil Universidade Federal da Bahia, Salvador, BA – Brazil

We are grateful in advance for the pertinent and constructive considerations and criticisms made of our work.^1^

Some of the limitations reported and cited in the letter were, albeit subtly, reported by the article's authors.

Table 1 provides other questions regarding the comparison (unpaired Student's T-test) between the groups regarding pharmacological therapy and the etiologies of heart failure.

**Table 1:** Comparison between groups WITH MEPS vs. WITHOUT MEPS regarding drug therapy and etiologies of patients undergoing ICD implantation as secondary prevention at Hospital Ana Nery

	With MEPS	Without MEPS	
	167	89	p
ACEI/ARB/ Sacubitril-valsartan	122 (73%)	67 (75%)	0.831
Beta blocker, n (%)	127 (76%)	76 (85.4%)	0.562
Spironolactone, n (%)	100 (60%)	47 (52.8%)	0.481
ISGLT2, n (%)	14 (8.3%)	11(12.3%)	0.323
Amiodarone, n (%)	99 (59.3%)	46 (51.7%)	0.761
Myocarditis, n (%)	18(10.7%)	9 (10.1%)	0.892
Idiopathic dilated	8 (4.8%)	7 (7.9%)	0.126
Amyloidosis	7 (4.2%)	5 (5.6%)	0.332
Peripartum	2 (1.2%)	4 (4.5%)	0.089
Non-compaction myocardium, n (%)	2 (1.2%)	2 (2.2%)	0.112

ACEI: angiotensin-converting enzyme inhibitor; ARB: aldosterone receptor blocker.

Table 2 describes the number of patients per group (WITH MEPS and WITHOUT MEPS) who died in the first year of implantation according to each etiology.

**Table 2:** Deaths < 1 year of patients WITH MEPS vs. WITHOUT MEPS according to the etiology of HF who underwent ICD implantation as secondary prevention of sudden cardiac death at Hospital Ana Nery

	Deaths with MEPS	Without MEPS
	10/167 (6%)	2/89 (2,2%)
Myocarditis	4 (40%)	─
Amyloidosis	1 (10%)	1 (50%)
Idiopathic dilated	3 (30%)	1 (50%)
Non-compaction myocardium	2 (20%)	─

We believe that some factors are associated with the low rate of patients using ISGLT2 in the sample, and we can mention some facts: our study was started 2.5 years before (2017) the publication of the study Dapagliflozin in Patients with Heart Failure and Reduced Ejection Fraction and the release for marketing of the drug, which was published in 2019. Another important factor addressed in the article is the low monthly family income of the sample and the high cost of the medication for the profile of the population studied, which may have contributed to one of the causes of low medication adherence (one of the economic and psychosocial markers - MEPS). It is also worth mentioning that, until the end of the study follow-up, there were no programs to facilitate free access to ISGLT2 in the time necessary that could eventually increase the rate of ISGLT2 use in time for data analysis.

We emphasize that despite drugs’ relevance in reducing mortality in patients with HF, such variables were not made available in the original version of the article; however, we believe that they were not a preponderant factor in changing the outcome studied.

Finally, there are no questions or concerns about the potential benefits of cardiac resynchronization therapy (CRT), given that our study did not address this prosthesis.

I remain at your disposal to clarify any further doubts.

William N. Carvalho
